# Performance of the dipstick screening test as a predictor of negative urine culture

**DOI:** 10.1590/S1679-45082017AO3936

**Published:** 2017

**Authors:** Alexandre Gimenes Marques, André Mario Doi, Jacyr Pasternak, Márcio dos Santos Damascena, Carolina Nunes França, Marinês Dalla Valle Martino

**Affiliations:** 1 Hospital Israelita Albert Einstein, São Paulo, SP, Brazil.; 2 Universidade de Santo Amaro, São Paulo, SP, Brazil.

**Keywords:** Bacteriuria/urine, Urinalysis/methods, Nitrites/urine, Sensitivity and specificity

## Abstract

**Objective:**

To investigate whether the urine dipstick screening test can be used to predict urine culture results.

**Methods:**

A retrospective study conducted between January and December 2014 based on data from 8,587 patients with a medical order for urine dipstick test, urine sediment analysis and urine culture. Sensitivity, specificity, positive and negative predictive values were determined and ROC curve analysis was performed.

**Results:**

The percentage of positive cultures was 17.5%. Nitrite had 28% sensitivity and 99% specificity, with positive and negative predictive values of 89% and 87%, respectively. Leukocyte esterase had 79% sensitivity and 84% specificity, with positive and negative predictive values of 51% and 95%, respectively. The combination of positive nitrite or positive leukocyte esterase tests had 85% sensitivity and 84% specificity, with positive and negative predictive values of 53% and 96%, respectively. Positive urinary sediment (more than ten leukocytes per microliter) had 92% sensitivity and 71% specificity, with positive and negative predictive values of 40% and 98%, respectively. The combination of nitrite positive test and positive urinary sediment had 82% sensitivity and 99% specificity, with positive and negative predictive values of 91% and 98%, respectively. The combination of nitrite or leukocyte esterase positive tests and positive urinary sediment had the highest sensitivity (94%) and specificity (84%), with positive and negative predictive values of 58% and 99%, respectively. Based on ROC curve analysis, the best indicator of positive urine culture was the combination of positives leukocyte esterase or nitrite tests and positive urinary sediment, followed by positives leukocyte and nitrite tests, positive urinary sediment alone, positive leukocyte esterase test alone, positive nitrite test alone and finally association of positives nitrite and urinary sediment (AUC: 0.845, 0.844, 0.817, 0.814, 0.635 and 0.626, respectively).

**Conclusion:**

A negative urine culture can be predicted by negative dipstick test results. Therefore, this test may be a reliable predictor of negative urine culture.

## RESUMO

**Objetivo:**

Verificar se a triagem de urina por fitas reativas é capaz de predizer a cultura de urina. Métodos Estudo retrospectivo realizado entre janeiro e dezembro de 2014 com 8.587 pacientes, com solicitação médica de triagem de urina (fita), sedimento urinário e cultura de urina.

**Foram analisados:**

sensibilidade, especificidade, valor preditivo positivo, valor preditivo negativo e curva ROC.

**Resultados:**

Foram positivas 17,5% das culturas. O nitrito apresentou sensibilidade de 28% e especificidade de 99%. O valor preditivo positivo foi de 89% e o valor preditivo negativo de 87%. Esterase apresentou sensibilidade de 79% e especificidade de 84%. Valor preditivo positivo e valor preditivo negativo foram de 51% e 95%, respectivamente. A combinação de nitrito ou esterase positivos apresentou sensibilidade de 85% e especificidade de 84%. Valor preditivo positivo e valor preditivo negativo foram, respectivamente, 53% e 96%. O sedimento positivo (mais de dez leucócitos por microlitro) apresentou sensibilidade de 92% e especificidade de 71%. O valor preditivo positivo foi 40% e o negativo, 98%. A combinação de nitrito e sedimento urinário positivos apresentou sensibilidade de 82% e especificidade de 99%. Os valores preditivos positivo e negativo foram 91% e 98%, respectivamente. Para o nitrito ou esterase positivos mais os leucócitos positivos, a sensibilidade foi de 94% e a especificidade de 84%. O valor preditivo positivo foi de 58% e o negativo foi de 99%. Com base na curva ROC, o melhor indicador de urocultura positiva foi a associação entre a esterase ou nitrito positivos na fita mais os leucócitos positivos no sedimento, seguido por nitrito e esterase positivos, sedimento urinário positivo isolado, esterase positiva isolada, nitrito positivo isolado e, finalmente, pela associação entre nitrito e sedimento urinário positivos (AUC: 0,845, 0,844, 0,817, 0,814, 0,635 e 0,626, respectivamente).

**Conclusão:**

Uma urocultura negativa pode ser prevista com resultados negativos na fita. Portanto, este teste pode ser um preditor confiável de urocultura negativa.

## INTRODUCTION

Urinary tract infection is one of the most common infections and often demands patient hospitalization.^1^ Urinalysis is the most requested screening test in patients with symptoms suggestive of urinary tract infection, such as dysuria, urinary incontinence and hematuria. This test analyzes urine biochemical and microscopic parameters, which may be altered in different pathological conditions.

Urinary biochemical parameters can be evaluated using the urine dipstick screening test. This test is thought to be an inexpensive and rapid diagnostic alternative,^2,3^ although its value has been questioned.^4,5^ The dipstick test detects urinary parameters such as glucose, protein, nitrite and leukocyte esterase (LE). The presence of nitrite and LE in urine may indicate infection, even though not all microorganisms have the ability to reduce nitrate to nitrite.^6^

Urinary sediment analysis using microscopy or digital flow morphology (system that auto-identifies and processes tube specimens by mixing, sampling, and analyzing urine particles automatically) is complementary to the dipstick test and contributes to the diagnosis of urinary tract infection. However, this test depends on several factors which can impact test results, such as sample collection, storage and transportation conditions, and technical expertise for accurate classification of elements of urinary sediment.^6-9^

Urine culture is the traditional gold standard for urinary tract infection diagnosis.^7,10,11^ However, this test is laborious and has a high turnaround time. Urine culture yields either positive (growth of more than 10^5^CFU/mL) or negative results. However, recent studies have shown that lower counts may also be significant in elderly and immunocompromised patients, since low colony counts may actually indicate urinary tract infection.^12^

In many countries urine culture requests are subject to strict criteria and algorithms to avoid unnecessary costs and labor. However, in Brazil, urinalysis and urine culture are frequently requested together despite the lack of appropriate indication.^13^

## OBJECTIVE

To investigate whether the urine dipstick screening test can be used to predict urine culture results.

## METHODS

A retrospective study was carried out between January and December 2014 using data collected from 8,587 patients seen in the emergency department. No exclusion criteria were adopted. All patients had the same medical request: urine screening (dipstick test), urinalysis (dipstick test and sediment analysis) and urine culture. Clinical features were not taken into account and data analysis was based exclusively on laboratory parameters. Positives urine cultures were associated with urinary tract infection or asymptomatic bacteriuria.

Patients were instructed to comply with urine sample collection instructions. Voided midstream urine samples were then collected. The dipstick screening test was performed in emergency department, while samples destined for urinalysis and urine culture were kept refrigerated at 2 to 8°C and sent to the central laboratory within 8 hours of collection. Analyses were performed by qualified well-trained professionals working on a 24x7-rotating schedule.

The dipstick screening test was conducted on semi-automated URYXXON 300™ (Macherey Nagel™, Germany) using Urofita 10 DLU (Macherey Nagel™, Germany). Urinalysis was performed on iRICELL™ (Iris Diagnostics, Beckman Coulter Company™, United States), a fully automated system integrating urine chemistry and microscopy, which combines Velocity™ (reflectance reading of urinalysis dipsticks) and iQ Sprint™ (digital flow morphology technology which isolates, identifies and characterizes particles based on flow-imaging microscopy). Quantitative urine culture analysis (10μL loop) was conducted using chromogenic media (CHROM CPS ID – bioMérieux™, France). Microorganism identification in positive samples was based on one of two methods: (1) direct identification based on colony color on culture media whenever possible, or (2) VITEK 2™ technology (bioMérieux™, France). When applicable, sensitivity tests were performed using VITEK 2™_._ Positive urine cultures were defined as those with growth greater than or equal to 10^5^CFU/mL of a single microorganism. Sensitivity, specificity, positive (PPV) and negative (NPV) predictive values were determined according to positive urine cultures.

The laboratory used in this study is accredited by College of American Pathologists; all quality control policies were applied to tests analyzed in this sample.

## RESULTS

Positive growth (≥10^5^CFU/mL) was observed in urine cultures of 1,604 out of 8,587 patients. The sample comprised 2,912 (33.9%) children (aged up to 12 years), 1,667 men (19.4%) and 4,008 women (46.7%). Average patient age was as follows: children, 4 years (median of 3 years); men, 45 years (median of 43 years) and women, 39 years (median of 37 years). A total of 1,732 (17.5%) out of 9,881 cultures requested were positive; *Gram*-negative bacilli were found in 1,590 (91.8%), *Gram*-positive cocci in 104 (6%) and yeasts in 38 (2.2%) cultures.

The Analisys of Variance (ANOVA) Bonferroni test (p<0.05) failed to reveal significant statistical differences regarding specificity (p=0.483), sensitivity (p=0.957), NPV (p=0.06) and PPV (p=0.618). Therefore, patients in this study were not stratified by age or gender and data grouped and analyzed as a single dataset. Microorganism distribution is presented in table 1.

**Table 1:** List of microorganisms found in urine samples

Microorganisms	n (%)
*Escherichia coli*	1,273 (73.5)
*Proteus mirabilis*	174 (10.0)
*Klebsiella pneumoniae*	72 (4.2)
*Staphylococcus saprophyticus*	52 (3.0)
*Enterococcus faecalis*	28 (1.6)
*Enterobacter aerogenes*	26 (1.5)
*Candida albicans*	25 (1.4)
*Citrobacter koseri*	16 (0.9)
*Streptococcus agalactiae*	16 (0.9)
*Enterobacter cloacae complex*	8 (0.5)
*Pseudomonas aeruginosa*	8 (0.5)
*Candida glabrata*	7 (0.4)
*Staphylococcus epidermidis*	5 (0.3)
*Proteus vulgaris*	3 (0.2)
*Candida parapsilosis*	3 (0.2)
*Candida tropicalis*	2 (0.1)
*Morganella morganii ssp morganii*	2 (0.1)
*Morganella morganii*	1 (0.1)
*Raoultella ornithinolytica*	1 (0.1)
*Enterobacter cloacae*	1 (0.1)
*Morganella morganii ssp sibonii*	1 (0.1)
*Staphylococcus aureus*	1 (0.1)
*Candida krusei*	1 (0.1)
*Staphylococcus hominis*	1 (0.1)
*Citrobacter freundii*	1 (0.1)
*Stenotrophomonas maltophilia*	1 (0.1)
*Enterococcus faecium*	1 (0.1)
*Escherichia hermannii*	1 (0.1)
*Haemophilus influenzae*	1 (0.1)

Total	1,732 (100)

Taken as a standalone parameter, nitrite had 28% sensitivity to predict positive urine cultures, with specificity of 99%, PPV of 89% and NPV of 87%. Similar analysis of LE revealed higher sensitivity (79%) of this parameter, but lower specificity (84%), with PPV and NPV values of 51% and 95%, respectively. Positive nitrite or LE dipstick test had the highest dipstick sensitivity (85%), with specificity of 84% and PPV and NPV of 53% and 96%, respectively.

Positive urinary sediment (10 leukocytes/μL) and positive urine cultures were also compared. The sensitivity of this parameter to predict positive cultures was 92%, with specificity of 71% and PPV and NPV of 40% and 98%, respectively.

The combination of positive nitrite test and positive urinary sediment had 82% sensitivity and 99% specificity, with PPV and NPV of 91% and 98%, respectively.

The last combination considered, positive nitrite or LE dipstick test and positive urinary sediment, showed the highest sensitivity (94%) and specificity (84%), with PPV and NPV of 58% and 99%, respectively (Table 2).

**Table 2:** Sensitivity and specificity of dipstick screening test and urinary sediment analysis to predict positive urine cultures, and respective positive and negative predictive values

	Dipstick (%)			Dipstick plus sediment (%)		Sediment (%)
		
	Nitrite+	Esterase+	Nitrite+ or esterase+	Nitrite+ leukocyte+	Esterase+ or nitrite+ and leukocyte+	Leukocyte+
Sensitivity	28	79	85	82	94	92
Specificity	99	84	84	99	84	71
PPV	89	51	53	91	58	40
NPV	87	95	96	98	99	98

PPV: positive predictive value; NPV: negative predictive value.

Negative dipstick test results with positive urine culture were observed in 267 (2.7%) cases. Of these, 226 (85%) were positive for *Gram*-negative bacilli, 28 (10%) for *Gram*-positive cocci and 13 (5%) for yeasts. Most patients with false negative dipstick test results and positive urine culture were women (206; 77%), followed by children (43; 16%) and men (18; 7%).

**Figure 1:**
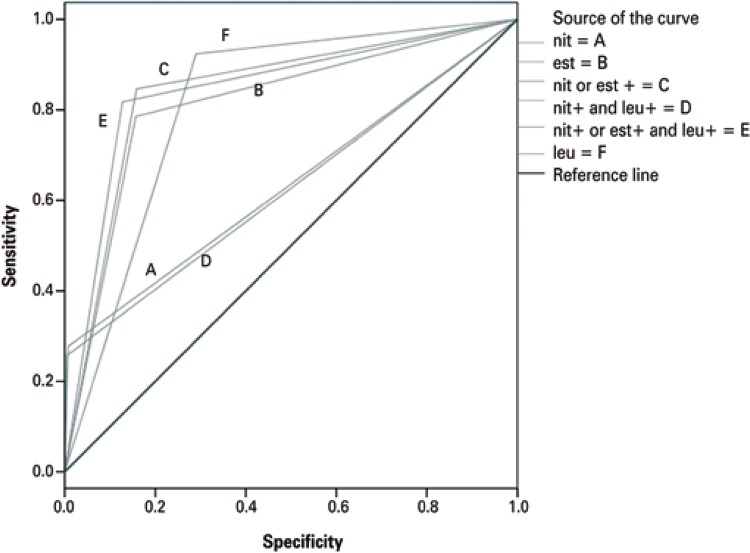
ROC curve

The Receiver Operator Characteristic (ROC) curve analysis revealed that the association of positive nitrite or LE tests and positive urinary sediment was the best indicator of positive urine culture (area under the curve – AUC: 0.845) (Figure 1). Other indicators were ranked in the following order: combination of positive LE or nitrite tests (AUC: 0.844), positive urinary sediment alone (AUC: 0.817), positive LE test alone (AUC: 0.814), positive nitrite test alone (AUC: 0.635) and finally combined positive nitrite test and positive urinary sediment (AUC: 0.626).

nit: nitrite; est: esterase; leu: leukocyte.

## DISCUSSION

Approximately 80% of the urine cultures in this study were negative. Similar data have been reported elsewhere.^14-17^In this analysis, positive cultures were defined as those with colony counts greater than 10^5^CFU/mL; fewer counts, from 10^4^CFU/mL^16,17^ or 10^3^CFU/mL,^18^have been reported in previous studies. A total of 632 cultures (6%) had growth between 10^2^ and 10^4^CFU/mL in this study. Urine cultures with less than 10^5^CFU/mL may indicate urinary tract infection in symptomatic women.^19-22^ Findings in this study are consistent with this view, given 84% of cultures with growth between 10^2^ and 10^4^CFU/mL were from female patients, the majority of them (77%) adults.

As in other studies, LE and nitrite were highly specific.^16,23-25^ Nitrite proved to be a low sensitivity indicator of positive urine culture in this study; similar data have been reported by Gieteling et al;^25^ following analysis of urine samples collected in ED settings (*i.e*., not first morning urine samples). The low sensitivity of nitrite was demonstrated in spite of growth of *Gram*-negative bacilli in 92% of positive cultures in this study. This may have reflected the fact that bacteria require a minimum of 4 hours to reduce the nitrate to nitrite^26^ or that not all *Gram*-negative bacilli contain nitrate reductase, the enzyme responsible for this conversion.

Different from other studies reporting high variability in LE sensitivity and specificity,^2,27,28^ the analysis of LE as a standalone parameter in this study suggested LE is more sensitive than nitrite (79% *versus* 27% sensitivity); still, it does not seem to be a good predictor of positive urine culture, given the low PPV (51%). On the other hand, LE was more reliable than nitrite for exclusion of potential urine culture orders (NPV of 95% compared to 87% of nitrite alone).

The combined analysis of nitrite and LE proved more sensitive than LE analysis alone (85% and 79% sensitivity, respectively), despite similar specificity (84%). The 96% NPV attributed to combined positive nitrite or LE tests suggests that urine culture requests can be ruled out in 96% of cases negative for both parameters, with significant time and cost saving for patients.

Our study also showed that the combination of sediment analysis and dipstick screening test is a good indicator of positive urine cultures, as previously reported.^25^

The association of urinary sediment analysis and dipstick screening test translated into significant improvements in sensitivity and NPV. Positive nitrite and LE tests combined with positive urinary sediment increased sensitivity from 85 to 94%, while maintaining specificity of 84%. Higher PPV and NPV were also observed when urinary sediment analysis and dipstick screening test results were combined, supporting data reported elsewhere.^25^

Based on results of this study, the combination of dipstick urine screening test and urinary sediment analysis is the best strategy to predict negative urine cultures. In this case, it is important to remember that urinalysis is a more laborious test which must be performed by trained professionals.

Nonetheless, this study revealed that urine cultures can be ruled out in 96% of cases with normal dipstick test results, supporting the use of this test as a valuable, economic and rapid alternative for urinary tract infection screening.^29^

Urine culture results in this study indicated that the dipstick test may give false negative results in 2.7% of cases, as previously reported.^30^

Despite the limitations in this study (*i.e.*, retrospective analysis of laboratory data), the large number of patients in the sample may provide valuable data to support the rational use of laboratorial tests, namely the avoidance of unnecessary laborious tests based on results of point of care urinary screening. Our findings are consistent with those of Humphries et al.,^31^ who emphasized the importance of rational request of laboratory tests, such as urine culture in patients suspected of urinary tract infection, given urine cultures are often requested in asymptomatic cases, leading to potentially inappropriate use of antimicrobials.

Data from this study support the significance of negative dipstick test results to rule out positive urine cultures. The performance of the urine dipstick screening test and conventional urinalysis were also compared and a good correlation observed.

## CONCLUSION

Results of this study showed that negative dipstick test results can be a good predictor of negative urine culture. Data also suggest that leukocyte esterase is a more reliable parameter than nitrite.

Clinical decisions based on dipstick urine screening tests could be both time and cost effective for patients, given negative results may eliminate the need for conventional urinalysis and urine culture. Rational laboratory test request policies are crucial, particularly in institutions and settings with limited resources.
